# Differences in own-face but not own-name discrimination between autistic and neurotypical adults: A fast periodic visual stimulation-EEG study

**DOI:** 10.1016/j.cortex.2023.10.023

**Published:** 2024-02

**Authors:** Annabel D. Nijhof, Caroline Catmur, Rebecca Brewer, Michel-Pierre Coll, Jan R. Wiersema, Geoffrey Bird

**Affiliations:** a Social, Genetic and Developmental Psychiatry Centre, Institute of Psychiatry, Psychology and Neuroscience, King's College London, UK; b Department of Experimental Clinical and Health Psychology. Ghent University, Belgium; c Department of Psychology, Institute of Psychiatry, Psychology and Neuroscience, King's College London, UK; d Department of Psychology, Royal Holloway, University of London, Egham, UK; e School of Psychology, Laval University, Quebec City, Canada; f Department of Experimental Psychology, University of Oxford, UK; g School of Psychology, University of Birmingham, UK

**Keywords:** Electroencephalography, Autism, Own name, Own face, FPVS

## Abstract

Self-related processing is thought to be altered in autism, with several studies reporting that autistic individuals show a diminished neural response relative to neurotypicals for their own name and face. However, evidence remains scarce and is mostly based on event-related potential studies. Here, we used EEG to measure the neural activity of autistic adults (20 for faces, 27 for names) and neurotypical adults (24 for faces, 25 for names) while they were watching rapidly alternating faces and names, through a relatively new technique called Fast Periodic Visual Stimulation. We presented strangers' faces or names at a base frequency of 5.77 Hz, while one's own, a close other's, and a specific stranger's face/name was presented at an oddball frequency of 1.154 Hz. The neurotypical group showed a significantly greater response to their own face than both close other and stranger faces, and a greater response for close other than for stranger faces. In contrast, in the autism group, own and close other faces showed stronger responses than the stranger's face, but the difference between own and close other faces was not significant in a bilateral parieto-occipital cluster. No group differences in the enhanced response to familiar names were found. These results replicate and extend results obtained using traditional electroencephalographic techniques which suggest atypical responses to self-relevant stimuli in autism.

## 1 Introduction

Humans have often been shown to have a strong bias for processing information that is self-related (Cunningham & Turk, 2017; Sui & Humphreys, 2015). This so-called self-bias is thought to be beneficial for social functioning, as a stronger sense of self is assumed to help one build more accurate models of the social world (Nijhof & Bird, 2019). Given the high social relevance of faces and names, and the high self-relevance of one's own face and name, the detection of one's own face or name among those of others is the most widely used measure of self-processing. For example, the processing of one's own face (Bortolon & Raffard, 2018; Gallup & Platek, 2021) and own name (Yang et al., 2013) has known reaction time advantages over those of familiar and unfamiliar others. In addition, fMRI studies show that seeing one's own face (Platek et al., 2008; Sugiura, 2015) as well as seeing or hearing one's own name (Carmody & Lewis, 2006; Tacikowski et al., 2013) result in distinct patterns of brain activation in comparison to other faces and names. For faces, one's own face evokes a distinct neural response even when compared to familiar faces (Bortolon & Raffard, 2018), but differences in the neural response to one's own name and that of a familiar other tend to be relatively small (Kotlewska & Nowicka, 2015; Tacikowski et al., 2011), demonstrating the importance of taking into account the familiarity of the faces and names used as comparison stimuli (Amodeo et al., 2023).

Considering the link between self-processing and social functioning, it is interesting to note that research suggests that autistic individuals,^1^ who are known to experience social difficulties (American Psychiatric Association, 2013), show indications of altered self-referential processing (Grisdale et al., 2014; Nijhof & Bird, 2019; Perrykkad & Hohwy, 2019). The evidence to date is mixed, however, especially at the behavioural level. For example, the recognition of one's own face in the mirror is intact in autistic children (Dawson & McKissick, 1984; Reddy et al., 2010), and several studies using various measures of self-bias found no differences between autistic and neurotypical individuals (Lind et al., 2019; Nijhof et al., 2022; Williams et al., 2017).

Differences in self-related processing in autism are found most consistently in event-related potential (ERP) studies of neural activity (Cygan et al., 2014; Nijhof et al., 2018, 2022; Nowicka et al., 2016). Cygan et al. (2014) studied responses to own and others' faces and visually presented names, and found that seeing one's own face or name resulted in larger P3 amplitudes compared to that of a close other in neurotypical, but not in autistic adults. The results for names were replicated in a second study, in which only name stimuli were included (Nowicka et al., 2016). Furthermore, Nijhof et al. (2018) observed an enhancement of the amplitude of the late parietal positivity for hearing one's own name in neurotypical adults, which was absent in autistic adults.

These findings make the electrophysiological neural processing of self-related stimuli a promising avenue for further study in autism. However, large-scale application of ERP studies in clinical populations is limited, as data collection is hindered by the noisy nature of EEG data. This results in the need for many trial repetitions and long recording times, which is not only burdensome for the person being tested but is also problematic as many ERP components are also sensitive to repetition (see e.g., Guillaume et al., 2009). Recording EEG during Fast Periodic Visual Stimulation (Heinrich et al., 2009; Rossion, 2014) may provide a means to counter this limitation of EEG research. Displaying visual stimuli at a fast, periodic frequency results in a neural response with a high signal-to-noise (SNR) ratio at the exact stimulation frequency (and its harmonics), since the broadband noise at other frequencies will not affect the signal of interest elicited by the stimuli. This high SNR response is known as the steady-state visual evoked potential.

In a recent FPVS-EEG advance, researchers present participants with two types of stimuli at two different stimulation frequencies (interspersing standard stimuli at a ‘base frequency’ with oddball stimuli at a different ‘oddball frequency’). This allows one to test whether the steady-state visual evoked potentials discriminate the property in which the oddball and standard stimuli differ, isolating this property from the lower-level processing of properties shared by both oddball and standard stimuli. It thus provides an ideal measure to identify specific aspects of face discrimination, such as discriminating between specific emotional expressions (Coll et al., 2019) or different facial identities (Liu-Shuang et al., 2014; Retter & Rossion, 2016; Yan & Rossion, 2020), respectively controlling for non-emotion- or non-identity-specific face-selective processes.

Because of the fast presentation rate and high SNR, one advantage of FPVS-EEG compared to the more widely used analysis of ERPs is that data can be acquired in just a few minutes, without the need for overt responses. This advantage makes FPVS-EEG especially suitable for testing individuals who cannot tolerate very long EEG recording sessions, such as young infants or certain clinical populations. The technique has already been used in autistic individuals, for example to demonstrate differences in emotion discrimination in autism (Van der Donck et al., 2019, 2020). Results for identity discrimination are mixed, however, with one study showing differences in individual (not self-) face discrimination in autistic children (Vettori et al., 2019), whereas a study in autistic adults found no differences (Dwyer et al., 2019).

To our knowledge, only two studies in neurotypical samples have compared responses to self-related and non-self-related material using FPVS-EEG, and none in autism. Kotlewska et al. (2017) used steady-state visual evoked potentials to show an enhanced response to a current photograph of a participant's own face, compared to a photograph of that participant when they were younger. However, they only presented stimuli of one category throughout a stimulus train, and thus did not investigate discrimination of own face stimuli when presented among other faces. In contrast, a recent study (Campbell et al., 2020) tested the recognition of one's own, a close other's and a stranger's identity when presented in a stream of other strangers' faces, in twelve neurotypical participants. Results showed that responses to the own face in occipito-temporal regions were reliably stronger than to both other types of faces, and stronger for the close other's face than for the stranger's face. Additionally, an identity-specific response was found for familiar faces (self and close other versus stranger faces) in a centro-parietal region.

The discrimination of own or other people's names has not yet been studied with FPVS-EEG. In general, there are relatively few studies that have used lexical items as stimuli in FPVS-EEG paradigms. Promisingly however, initial findings suggest that participants can reliably distinguish between words of different semantic categories (Volfart et al., 2021), as well as between words and pseudowords (Barnes et al., 2021; Lochy et al., 2015) when these are presented at the fast rates used in FPVS-EEG.

In the current study, FPVS-EEG was used as a means to test further the hypothesis that self-related processing, as compared to other-related processing, is diminished at the neural level in autism. More specifically, FPVS-EEG was used to study the discrimination of one's own face and name (as compared to those of a close other and a stranger), in autistic and neurotypical adults. Usually, face and name processing are studied in isolation, but here both stimulus types were combined within the same sample to allow for direct comparison between own name and own face processing. In order to do so, a task design similar to that of Campbell et al. (2020) was used to study face processing, with the addition of a second task in which first names for the same three conditions (self, close other, stranger) were presented as oddball stimuli, to test for their discrimination among strangers' first names. Participants passively viewed the face and name stimuli, with an accompanying behavioural task serving only as an attention check. To maximize stimulus variability and thus ensure that we were measuring identity discrimination rather than discrimination on the basis of low-level stimulus properties (Coll et al., 2019), various naturalistic photographs were used as stimuli for the face task, and font, size and capitalization varied for the name task. We hypothesized that neural responses to faces would show self-specific as well as familiarity effects, particularly in centro-parietal and bilateral occipito-temporal regions, in line with Campbell et al. (2020). For names, self-specific (Cygan et al., 2014; Nijhof et al., 2018) as well as familiarity effects (Kotlewska & Nowicka, 2015; Tacikowski et al., 2011) could also be expected based on previous literature. Importantly, enhanced responses to the self-related stimuli were expected to be diminished (or even absent) in autistic individuals (Cygan et al., 2014; Nijhof et al., 2018; Nowicka et al., 2016).

## 2 Methods

### 2.1 Participants

Initially, 31 autistic adults and 27 adults without any psychiatric or neurological diagnosis were recruited. However, one participant from the autism group had to be excluded due to failing the attention checks. Further, ten autistic participants and three neurotypical participants were unable, or chose not to, provide the images that were needed for the Face task. In addition, due to technical issues or too many noisy channels (>10 %, i.e., ≥ seven channels), data on the Name task could not be analysed for three autistic participants and two neurotypical participants. Hence, the final samples for the Face task consisted of 20 autistic and 24 neurotypical participants (of whom 19 and 23 also provided data on the Name task: the combined sample across both tasks), and the final samples for the Name task consisted of 27 autistic and 25 neurotypical participants. Additional demographic information can be found in Table 1, Table 2, Table 3. In the main text, only the results for the combined sample are reported. However, results for the full samples of the Face and Name task separately are provided as Supplementary Material. Note that for the final samples, there was a significant difference in age between groups. However, age was found to be unrelated to the main comparison of interest (Self – Close Other) for all clusters (all *r* < .18, all *p*-values >.22), and therefore age was not entered as a covariate in the analyses.

**Table 1:** Demographics of the final autism and neurotypical (NT) samples across both tasks.

	Autism (*N* = 19, 9 male) M (SD)	NT (*N* = 23, 8 male) M (SD)	*t*, *p* values
Age	39.2 (12.7)	27.8 (7.0)	3.47, *p* = .002
AQ	36.4 (6.5)	15.4 (4.6)	12.25, *p* < .001
WASI-II	113.4 (18.6)	107.3 (8.7)	1.19, *p* = .25

AQ = Autism Spectrum Quotient, WASI-II = Wechsler Abbreviated Scale of Intelligence.

**Table 2:** Demographics of the final autism and neurotypical (NT) samples for the Face task.

	Autism (*N* = 20, 10 male) M (SD)	NT (*N* = 24, 9 male) M (SD)	*t*, *p* values
Age	39.0 (12.4)	28.1 (7.0)	3.49, *p* = .002
AQ	36.3 (6.4)	15.2 (4.6)	12.15, *p* < .001
WASI-II	112.8 (18.1)	107.0 (8.6)	1.17, *p* = .26

AQ = Autism Spectrum Quotient, WASI-II = Wechsler Abbreviated Scale of Intelligence.

**Table 3:** Demographics of the final autism and neurotypical (NT) samples for the Name task.

	Autism (*N* = 27, 15 male) M (SD)	NT (*N* = 25, 8 male) M (SD)	*t*, *p* values
Age	39.6 (12.7)	27.4 (6.9)	4.35, *p* < .001
AQ	37.2 (6.1)	15.9 (5.7)	12.48, *p* < .001
WASI-II	111.6 (19.2)	106.9 (8.4)	1.06, *p* = .30

AQ = Autism Spectrum Quotient, WASI-II = Wechsler Abbreviated Scale of Intelligence.

Autistic participants were recruited through an existing database of research volunteers, and neurotypical participants through King's College London e-mail recruitment and social media. All participants had normal or corrected-to-normal vision. Autistic participants were included on the basis of a DSM-IV-based autism-related diagnosis, or a diagnosis of Autism Spectrum Disorder (DSM-5). For 21 participants, this diagnosis was verified with the Autism Diagnostic Observation Schedule (ADOS-2; Lord et al., 2012), Module 4, by a trained psychologist. Seven participants could not be tested on the ADOS-2 due to the onset of the Covid-19 pandemic. However, excluding them from the analyses did not significantly alter our main findings, and they did not show lower scores (M: 40.9, SD: 6.3) than the other autistic participants on the Autism Spectrum Quotient (Baron-Cohen et al., 2001; https://embrace-autism.com/autism-spectrum-quotient). Both autistic and neurotypical participants completed this questionnaire as a measure of self-reported autism traits, and the autistic participants reported a significantly higher level of autistic traits. Participants in both groups performed the Vocabulary and Matrix Reasoning subtests of the Wechsler Abbreviated Scale of Intelligence (WASI-II; Wechsler, 2011), with the estimated IQ scores indicating that the groups did not differ in intelligence (see Table 1, Table 2, Table 3 for statistical comparisons of groups). Legal copyright restrictions prevent public archiving of the WASI-II, which can be obtained from the copyright holders in the cited reference. All participants provided written informed consent prior to the study and received financial compensation. The study was approved by the ethics committee of Royal Holloway University of London (UDJT023). The conditions of our ethics approval do not permit public archiving of the study data. Readers seeking access to the data should contact the lead author. Access will be granted to named individuals in accordance with ethical procedures governing the reuse of sensitive data.

### 2.2 Procedure

Participants took part in a single session of approximately 90 min. After preparation for recording, EEG activity was recorded during three tasks, which were presented in random order across participants: the Face task and Name task described here, and another unrelated task (in which participants viewed screens of varying colours) that will be reported elsewhere. After the EEG session, participants completed the AQ questionnaire, and completed the WASI-II subtests if no IQ estimate was already available. For the autistic participants, if no ADOS had been performed then this was completed in a separate test session.

### 2.3 EEG recording

EEG data were acquired from a 64-channel BrainAmp DC-coupled recording system using Brain Vision Recorder software (Brain Products, Gilching, Germany). Active electrodes were mounted into the ActiCap following a 10–20 montage, with a ground electrode at AFz and a reference electrode at FCz. The sampling rate was 500 Hz, and impedances were kept below 15 kΩ.

### 2.4 Task

**Fig. 1:**
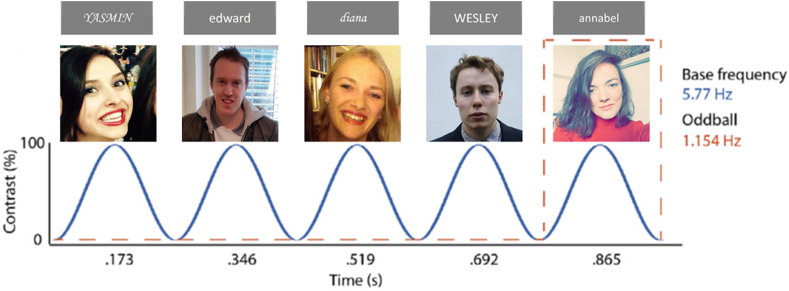
Graphical representation of one cycle on the Name task (top)/Face task (bottom), with every fifth name or face (i.e., the oddball) belonging to the same identity (Self, Close Other or Stranger).

To measure participants’ ability to discriminate self- and other-related stimuli, we used an FPVS-EEG paradigm in which the self- and other-related stimuli were presented as oddballs in a baseline sequence consisting of stranger stimuli (see Fig. 1 for an illustration of the paradigm; task code can be found at https://osf.io/3mn2t). All stimuli were presented on a grey background, on a 17-inch LCD monitor with a measured refresh rate of 57.7 Hz. Each train consisted of 96 cycles of five faces/names and thus lasted 83.2 sec in total, and the order of the different tasks (Face/Name task), as well as the different trains within each task (Self/Close Other/Stranger) were randomized. Within each train, every fifth face/name was always an oddball stimulus of the same identity. Stimuli were presented at a base frequency of 5.77 Hz, and thus the oddball frequency was 1.154 Hz.

To ensure that participants were attentive to the stimuli being presented, they were asked to fixate on a black fixation cross that was presented centrally throughout the entire stimulus train (for faces, this roughly corresponded to the area just above the nose; names were presented just above the fixation cross). This cross changed colour from black to red at ten random moments during each train, for a duration of 333 ms. Participants were instructed to press the left mouse button as quickly as possible when this happened.

### 2.5 Face stimuli

To ensure any effects observed at the oddball frequency could not be explained by each fifth face being perceptually identical (Coll et al., 2019), ten different images were used for each of the oddball conditions (self, close other, stranger). Participants were asked to provide ten naturalistic images of themselves, and ten of a person close to them. The requirements for the images were that these were in full colour, taken no more than eight years ago, and that their full face was visible (without any (sun)glasses). In both groups, eleven participants selected a family member as their ‘close other’. Ten NT participants and five autistic participants selected their partner. Seven NT participants and nine autistic participants selected a friend. The remaining participants chose not to disclose their relationship to the person in the images. Ten images of another randomly selected participant in the study were used for the stranger condition. There was no effect of Condition (Self, Close Other, Stranger; *p* = .87) or Condition × Group interaction (*p* = .48) on the average luminance of images, and differences in luminance between conditions were not found to correlate with neural differences. The distractor images used for the base frequency were randomly selected from a database containing 306 naturalistic images: three images for 102 different identities (51 male, 51 female; note that our ethical approval does not allow the reuse of face stimuli beyond the current experiment), composed of consenting acquaintances of the researchers. The only constraint was that no two images of the same identity were ever presented consecutively. Both oddball and distractor images were cropped to 300 × 300 pixels, containing only a single face. The exact size of presentation was then varied within a range of 90 % to 110 % of the original size. Further, to create smoother transitions between the different images, the contrast of the faces was sinusoidally modulated.

### 2.6 Name stimuli

Participants were asked to provide their own first name, and that of the close other person whose images they had selected. The name of another study participant, with which they reported having no personal association, was randomly selected for the stranger condition. The average number of digits of the names (Self: 5.9 (SD: 1.7); Close Other: 5.4 (SD: 1.5); Stranger: 5.7 (SD: 1.2)) did not differ significantly between conditions, neither within nor across groups (all *p*-values >.09). To ensure any effects observed at the oddball frequency could not be explained by each fifth name being perceptually identical (Coll et al., 2019), four stimuli for each first name were created: using all capitals versus no capitals, and using two different fonts (Calibri and Monotype Corsiva, size 40). The distractor images presented at the base frequency were randomly selected from a list containing 96 first names (48 male, 48 female), composed from two databases of common first names in the United Kingdom (BabyCentre, 2000; UK Office for National Statistics, 2014). The list contained these 96 names in the same four variations of capitalisation and font (see https://osf.io/3mn2t/for these stimuli). The only constraint was that no two variations of the same name were ever presented consecutively. All names were presented in white on a grey background.

### 2.7 EEG data pre-processing and analysis

Pre-processing was performed with the Letswave toolbox (https://github.com/NOCIONS/letswave6) running on MatLab R2020a (MathWorks). No analysis code was used. Data were first re-referenced against the average of all electrodes, and any bad channels (no more than two per person) were interpolated. Subsequently, a band-pass filter of .1–70 Hz was applied. Data were then segmented into three epochs of 84 sec (one per condition), and ocular correction was performed on each of these epochs through Independent Component Analysis, using the *Runica* algorithm and a square matrix. An average of 1.7 components was removed (SD: .9, range: 0–6). Epochs were then further cropped to contain an integer number of 1.154 Hz cycles (96 cycles of 1/1.154 *s*ec ≈ 83.2 sec). A Fast Fourier Transform was applied to transform the data for each electrode from the time domain to the frequency domain, while normalizing the amplitudes (N/2).

Amplitude data around the oddball frequency and its harmonics were extracted with a frequency resolution of .012 around the oddball frequency (1.142–1.166 Hz), and its first seven harmonics, not including the fifth as this is the baseline frequency (i.e., six harmonics: 2.31, 3.46, 4.62, 6.92, 8.08, 9.23 Hz). The number of harmonics to include was calculated on the basis of z-scores of the grand average across all electrodes, participants and conditions, where a harmonic was considered significant if *Z* > 2.32, i.e., *p* < .01 (Retter & Rossion, 2016). To compare the oddball and harmonics frequencies to the surrounding noise, signal-to-noise subtracted amplitudes were computed by extracting the average of the 20 frequency bins (±.12 Hz) surrounding the oddball or one of the harmonic frequencies (Rossion, 2014), excluding the two bins immediately adjacent to the bins of interest, in order not to include any potential spread of the signal in the correction (Norcia et al., 2015). This average was then subtracted from the frequency bin of interest. Statistical analyses of the baseline-subtracted SNR data were performed using RStudio (version 1.2.5033; see https://osf.io/3mn2t for code) for selected regions of interest (ROIs).

**Fig. 2:**
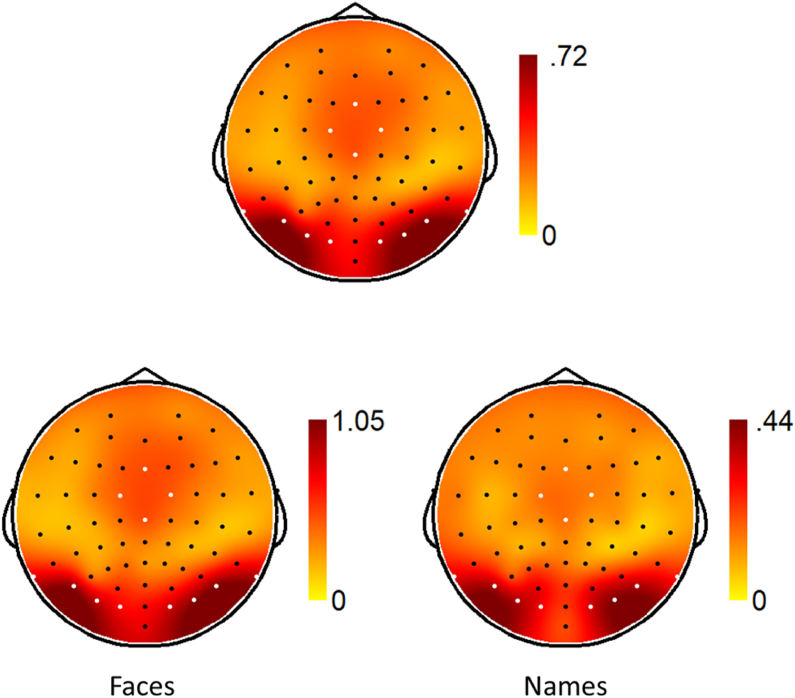
Scalp topography for the baseline-subtracted amplitudes at the oddball frequency (1.154 Hz) and the first six harmonics, across all participants and conditions. Scale is set to the maximum amplitude for each image. Electrodes selected for analysis are highlighted in white. Top: Across Face and Name tasks. Bottom left: Face task. Bottom right: Name task.

**Fig. 3:**
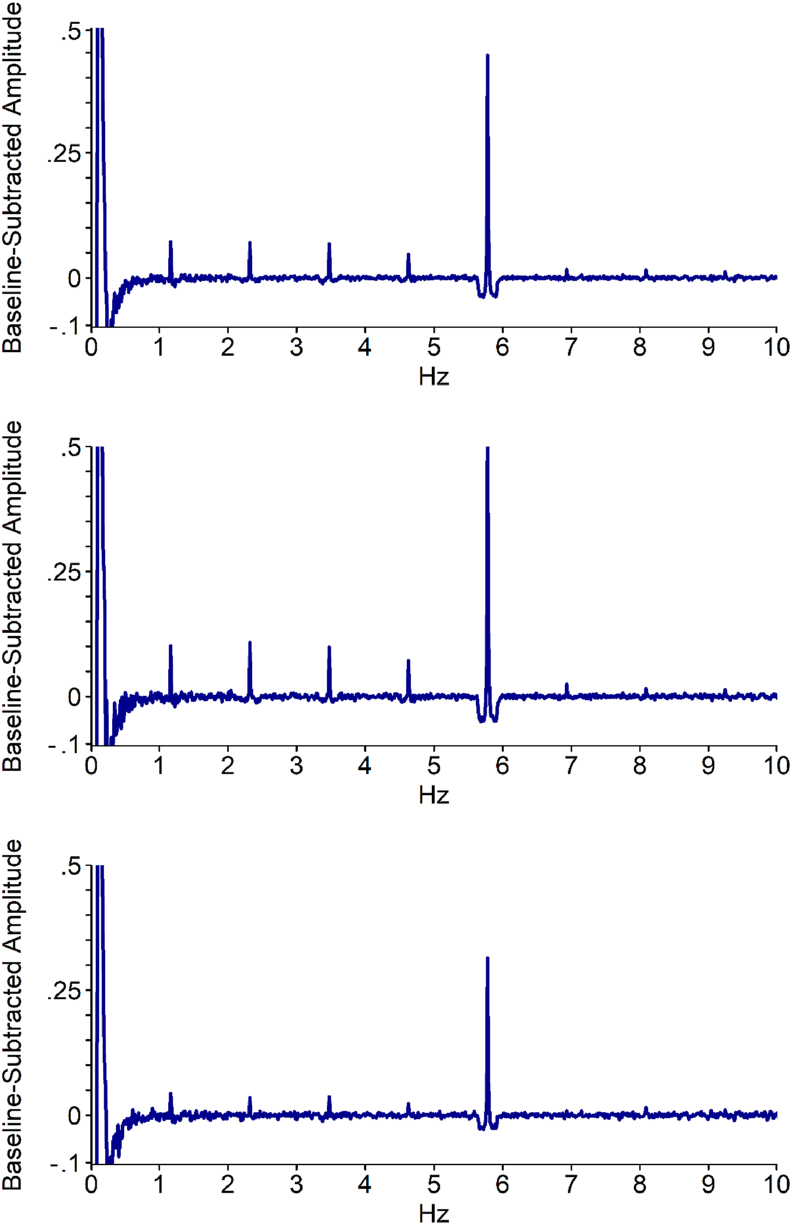
Baseline-subtracted amplitude for each frequency bin, across both groups and across all electrodes and conditions. Note that the .1 Hz peak is an effect of the bandpass filter. Top: Across Face and Name tasks. Middle: Face task. Bottom: Name task.

**Fig. 4:**
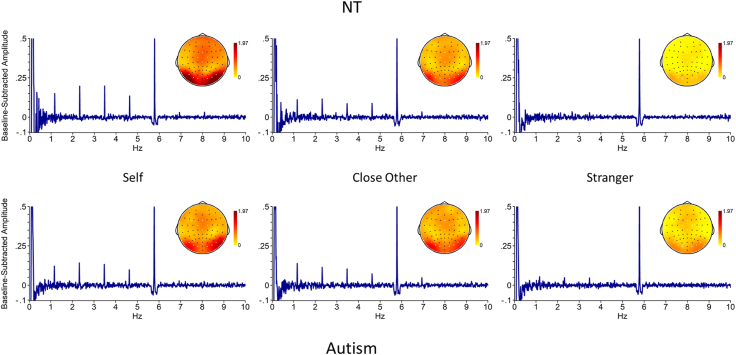
Face task: Baseline-subtracted amplitude for each frequency bin, and scalp topography, per group and per condition. Note that the .1 Hz peak is an effect of the bandpass filter. Electrodes selected for analysis are highlighted in white on the scalp topography. Top: neurotypical (NT) group, bottom: autism group.

**Fig. 5:**
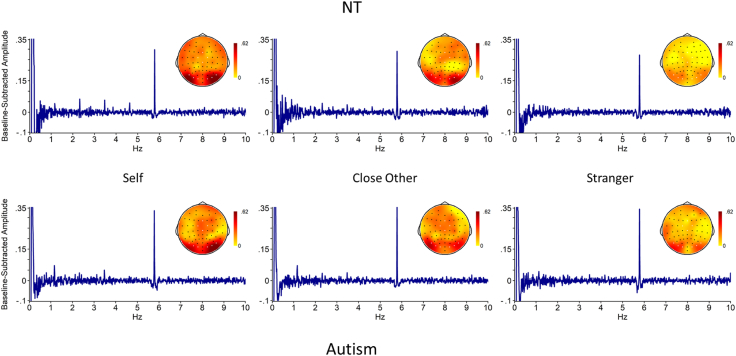
Name task: Baseline-subtracted amplitude for each frequency bin, and scalp topography, per group and per condition. Note that the .1 Hz peak is an effect of the bandpass filter. Electrodes selected for analysis are highlighted in white on the scalp topography. Top: neurotypical (NT) group, bottom: autism group.

ROIs were selected based on visual inspection of the scalp topography of the oddball baseline-subtracted responses across participants, conditions, and stimulus types (collapsed localizer approach; Luck & Gaspelin, 2017), which resulted in three ROIs. The four electrodes with maximum response were chosen to quantify the activity in these ROIs. Scalp topographies are displayed, across the two tasks and for both tasks separately, in Fig. 2. For visualization of the baseline-subtracted SNR across tasks (Fig. 3) and on both tasks (Fig. 4, Fig. 5), we computed the baseline-subtracted amplitudes for each frequency bin, across groups, conditions and electrodes.

The main analysis focused on a left (P7, PO7, O1, TP9) and right (P8, PO8, O2, TP10) parieto-occipital cluster, as well as a frontocentral cluster (Fz, FC1, FC2, Cz), and was carried out using IBM SPSS version 29. No analysis code was used. A mixed-model ANOVA was performed on the extracted data for each cluster, with within-subjects factors Stimulus Type (Faces, Names), Condition (Self, Close Other, Stranger) and between-subjects factor Group (Autism, Neurotypical). Baseline-subtracted amplitudes in the left and right parieto-occipital clusters were tested within the same ANOVA, adding a Laterality factor (Left, Right). Significant interactions were followed-up per stimulus type, and with two-tailed paired-samples as well as between-samples *t*-tests. It is reported if these do not survive Bonferroni correction for multiple comparisons. Further, measures of effect size are reported: partial eta squared (η_p_^2^) values for the ANOVAs and Cohen's d or dz for *t*-tests (Cohen, 1988). In cases where the assumption of sphericity was violated according to Mauchly's test, a Greenhouse-Geisser correction was applied, and corrected values are reported.

## 3 Results

### 3.1 Attention check

Average accuracy on the attention check was 95.1 % (SD: 6.7 %). There was a significant effect of Stimulus Type (*F*(1,40) = 19.00, *p* < .001, ηp2 = .32), with accuracy on the Name task (99.1 %) being higher than on the Face task (90.8 %). In addition, there was a significant Stimulus Type × Group interaction (*F*(1,40) = 4.43, *p* = .04, ηp2 = .10), with accuracy on the Face task being significantly higher (t(40) = 1.76, *p* < .05, *d* = .56) in the neurotypical group (94.3 %) than in the autism group (87.2 %), with no significant difference in accuracy (98.7 %; 99.6 %) on the Name task (t(40) = −.96, *p* = .35, *d* = .31). However, there were no significant main or interaction effects of Condition (all *p*-values >.29), nor a main effect of Group (*p* = .13).

### 3.2 Face and name discrimination

The ANOVA for the parieto-occipital clusters revealed a significant main effect of Stimulus Type (*F*(1, 40) = 124.38, *p* < .001, ηp2 = .75), with baseline-subtracted amplitudes being significantly higher for faces than for name stimuli. The only effect of laterality was a significant Laterality × Condition interaction (*F*(2, 80) = 4.42, *p* = .02, ηp2 = .10), with baseline-subtracted amplitudes for the Self condition being larger in the right than in the left cluster (*p* = .04), with no such difference for the Close Other or Stranger conditions (*p* = .46 and *p* = .49, respectively). There was a significant main effect of Condition (*F*(2, 80) = 46.46, *p* < .001, ηp2 = .54), as well as a significant Condition × Group interaction (*F*(2, 80) = 4.95, *p* = .009, ηp2 = .11). No other main or interaction effects reached significance, except the Stimulus Type *x* Condition × Group interaction (*F*(2, 80) = 3.81, *p* = .03, ηp2 = .09). We decomposed this interaction by performing ANOVAs for the Face and Name tasks separately.

For the Face task, there was a significant main effect of Condition (*F*(2, 80) = 44.75, *p* < .001, ηp2 = .53), with baseline-subtracted amplitudes in the Self condition (*M* = 1.37, SE = .10) being significantly higher than for both the Close Other (*M* = .93, SE = .08, *p* < .001) and Stranger (*M* = .36, SE = .05, *p* < .001) conditions, and higher for Close Other than for Stranger (*p* < .001). Furthermore, the interaction Condition *x* Group was also significant (*F*(2, 80) = 5.76, *p* = .005, ηp2 = .13) – the difference between Self and Close Other was found to be significantly larger in the Neurotypical group than in the Autism group (t(40) = 2.60, *p* = .01, *d* = .82), as was the difference between Self and Stranger (t(40) = 3.03, *p* = .004, *d* = .96). The difference between Close Other and Stranger did not differ between groups (t(40) = .75, *p* = .46, *d* = .24). Finally, there was a significant Laterality *x* Condition effect, driven by the difference between Self and Stranger being larger in the right than in the left cluster (t(41) = −2.04, *p* < .05, *d* = .31).

For the Name task, the main effect of Condition was also significant (*F*(2, 80) = 7.64, *p* < .001, ηp2 = .16). Baseline-subtracted amplitudes were significantly higher for the Self (*M* = .46, SE = .05) than for the Stranger condition (*M* = .22, SE = .04, *p* = .001) and for the Close Other (*M* = .38, SE = .04) than for the Stranger condition (*p* = .01). The difference between Self and Close Other was not significant (*p* = .16). The Condition × Group interaction was not significant (*F*(2, 80) = .54, *p* = .59, ηp2 = .01). Further, there were no significant main or interaction effects of Laterality (all *p*-values >.18).

The ANOVA for the frontocentral cluster also revealed a significant main effect of Stimulus Type (*F*(1, 40) = 62.06, *p* < .001, ηp2 = .61): baseline-subtracted amplitudes were higher for faces than for names. Further, the main effect of Condition was significant (*F*(2, 80) = 28.94, *p* < .001, ηp2 = .42), as was the interaction effect of Stimulus Type and Condition (*F*(2, 80) = 8.64, *p* < .001, ηp2 = .18). Splitting the data up by Stimulus Type, it was found that for face stimuli, baseline-subtracted amplitudes in the Self condition (*M* = .70, SE = .06) were larger than in the Close Other condition (*M* = .53, SE = .06; *p* = .02) and the Stranger condition (*M* = .20, SE = .04; *p* < .001). The difference between the Close Other and Stranger condition was also significant (*p* < .001). For name stimuli, there was no significant difference between Self (*M* = .21, SE = .03) and Close Other stimuli (*M* = .20, SE = .04; *p* = .94). However, the differences between Self and Stranger (*M* = .07, SE = .03; *p* = .002) and Close Other and Stranger (*p* = .01) were significant. Finally, for the frontocentral cluster there were no significant main or interaction effects of Group (all *p*-values >.09).

As mentioned, data from both tasks were not available for all participants. Results of the separate analyses for the full samples on the Face and Name task are provided as Supplementary Material. Importantly, these analyses did not alter the conclusions drawn from the cross-task analysis described above.

## 4 Discussion

The current study provided the first investigation of own-face and own-name discrimination in autism using FPVS-EEG. Results showed that both autistic and non-autistic adults showed significant neural responses to specific personal identities that were presented as oddballs among identities belonging to random strangers, for both faces and names. This was evident from neural data obtained in less than 5 min. Moreover, responses were more robust when the stimuli were familiar, with responses being strongest for distinguishing one's own face, and familiar names, from those of strangers. Neurotypical adults showed both a self-specific and a familiarity effect for faces (own > close other > stranger face), and a familiarity effect for names (both own and close other > stranger name). For the autistic adults, familiarity effects were also found for both faces and names, but they showed a reduction in the specifically enhanced response to their own face.

Both faces and names elicited signals with high SNRs in bilateral parieto-occipital clusters, similar to the regions typically associated with face recognition (Liu-Shuang et al., 2014; Retter & Rossion, 2016; Yan & Rossion, 2020), as well as in an additional frontocentral cluster (Campbell et al., 2020).

Interestingly, and in line with our hypothesis, for the face task, the autism group showed no significant differences between their own and close other's faces in the selected parieto-occipital clusters, in contrast to the neurotypical adults, although there was no group difference in the responses in the frontocentral cluster. The difference between one's own and close other's faces was less pronounced in the autism group in both left and right parieto-occipital clusters, despite the self-specific response in the neurotypical group being stronger in the right hemisphere. Our study thus extends previous findings of a diminished response to seeing one's own face compared to a familiar other's face in autistic adults, as compared to neurotypical adults (Cygan et al., 2014; Lu et al., 2015). As familiarity processing was intact, this group difference appears to be self-specific. We thus provide further evidence for altered self-referential processing in autism, utilizing the high SNR of FPVS-EEG. Furthermore, the fact that the group difference was self-specific corroborates the results of Dwyer and colleagues (Dwyer et al., 2019), who investigated the perception of unfamiliar face identities using FPVS-EEG and found no differences between autistic and neurotypical adults.

In contrast to the findings for neural responses to faces, no group differences were found on the name task. Both neurotypical and autistic adults only showed a familiarity effect in their neural responses: stronger responses for own and close other's names than for stranger's names in left and right temporo-parietal regions. The lack of a self-specific response to names (and the corresponding lack of a group difference) contrasts with earlier reports of stronger neural responses to one's own name as compared to close other names in neurotypical individuals, and of this difference being diminished in autism (Cygan et al., 2014; Nijhof et al., 2018; Schwartz et al., 2020). However, using fMRI, Tacikowski et al. (2011) reported that neural responses to own and close other names are highly similar (with the exception of increased activity in the Inferior Frontal Gyrus for the own name). Potentially, these mixed findings could in part be explained by the suggestion that for names, self-specific effects only appear at later stages of self-processing (Nijhof et al., 2018), and that it may be dependent on the specific presentation modality (with stronger effects for auditory presentation of names), paradigm and analysis method, whether or not such late-stage effects can be detected (Williams et al., 2017). More generally, it may be that the passive viewing of task-irrelevant names, together with the visual presentation of the task-relevant fixation cross just below them, may have cued attention away from the names, resulting in overall weaker responses to the names than to the faces. This is unlikely however, as we did observe significant differences between familiar names and stranger names, and the attention check data suggested that names were less distracting than faces.

It should be noted that there was an age difference between the autism and non-autism groups in our study. However, note that all participants were adults, and that age was shown not to relate significantly to the main comparison of interest (Self – Close Other) for any cluster, and can thus not explain the group difference between these two conditions in the response to faces. A further limitation is that seven autistic adults could not be tested on the ADOS-2, but encouragingly, excluding the adults for whom no ADOS score was available from the analyses did not significantly alter any of the findings, and they did not show lower scores than the other autistic participants on the Autism Spectrum Quotient. In addition, no a priori power analysis was carried out to determine the sample size, which would have been valuable. However, the study's sample size was in line with typical sample sizes in FPVS-EEG studies.

Finally, we did not include a measure of individuals’ perceived closeness to the close other or stranger. This could be relevant as familiarity with the close other may vary between individuals, because these were chosen freely by the participants themselves. It could be argued that the close others were experienced to be relatively less close to the participants in the autism group, given the reported differences in friendship quality and frequency of contact in autistic individuals (Petrina et al., 2014). However, no differences in perceived closeness to self-selected close others were found between autistic and neurotypical individuals in previous studies (Nijhof et al., 2020, 2022). More importantly, even if such a difference were present between our current samples, this would predict a larger difference between self and close other in autism, instead of a smaller one.

More generally, the results of this study emphasize the utility of FPVS-EEG for studying the discrimination of faces and names in clinical groups, and the possibilities of the technique for further studies of neural processing in these groups. The fact that data can be acquired in a short amount of time (in this study, time on each of the two tasks was less than 5 min), without requiring any overt responses, makes FPVS-EEG a tool with excellent potential. It can be used to reliably test various populations that may otherwise be challenging to study, such as infants, and importantly also minimally or nonverbal individuals, whom current research largely overlooks (Happé & Frith, 2020).

In conclusion, using FPVS-EEG, we managed to extend previous findings of a diminished response to seeing one's own face in autistic adults as compared to neurotypical adults. This effect appears to be self-specific and could not merely be attributed to familiarity, as the two groups did not differ in their response to close other faces, and both showed a heightened response to familiar names.

## Author statement

Annabel D. Nijhof: Conceptualization; Data curation; Formal analysis; Funding acquisition; Investigation; Methodology; Visualization; Writing - original draft.

Caroline Catmur: Conceptualization; Methodology; Supervision; Writing - review & editing.

Jan R. Wiersema: Supervision; Writing - review & editing.

Rebecca Brewer: Methodology; Writing - review & editing.

Michel-Pierre Coll: Methodology; Formal analysis; Writing - review & editing.

Geoffrey Bird: Conceptualization; Funding acquisition; Methodology; Supervision; Writing - review & editing.

## Declaration of competing interest

None.

## Footnotes

1 To respect the wishes of autistic individuals and to use standard scientific parlance, we will henceforth use the term ‘autistic’, a term endorsed by many individuals with ASD in the United Kingdom (Kenny et al., 2016).
