# Supplementary material for: Differences in own-face but not own-name discrimination between autistic and neurotypical adults: A fast periodic visual stimulation-EEG study
Source: Cortex. 2024 Feb;171:308–18. doi: 10.1016/j.cortex.2023.10.023 (PMC11068592; doi:10.1016/j.cortex.2023.10.023)
Supplement: Multimedia component 1 [file mmc1.docx]

**Supplementary Material**

**Face task**

***Attention check***

Average accuracy on the attention check was 91.5% (SD: 12.6%) with no significant differences between groups (t(42) = 1.72, p = .10) or conditions (all p-values > .67).

***Face discrimination***

The ANOVA on the two parieto-occipital clusters revealed a significant main effect of Condition (F(2, 84) = 45.52, p < .001, η_p_^2^ = 0.52), with baseline-subtracted amplitudes in the Self condition (M = 1.35, SE = 0.10) being significantly higher than for both the Close Other (M = .90, SE = 0.08; p < .001) and Stranger (M = 0.35, SE = 0.05; p < .001) condition, and higher for Close Other than for Stranger (p < .001). Furthermore, the interaction with Group was also significant (F(2, 84) = 7.13, p = .001, η_p_^2^ = 0.15) – the difference between Self and Close Other was found to be significantly larger in the Neurotypical group than in the Autism group (t(42) = 2.93, p = .006, d = 0.87), as was the difference between Self and Stranger (t(42) = 3.35, p = .002, d = 0.99). The difference between Close Other and Stranger did not differ between groups (t(42) = 0.79, p = .43, d = 0.24). There were no significant main or interaction effects with Laterality (all p-values > .05).

We decomposed the significant Condition x Group interaction by performing the repeated-measures ANOVA separately within each group. In the Neurotypical group, the effect of Condition was significant (F(2, 46) = 63.42, p < .001, η_p_^2^ = 0.73), with baseline-subtracted amplitudes in the Self condition (M = 1.64, SE = 0.13) being significantly higher than for both the Close Other (M = 0.90, SE = 0.11; p < .001) and Stranger (M = 0.27, SE = 0.06; p < .001) condition, and higher for Close Other than for Stranger (p < .001). There were no significant main or interaction effects of Laterality (p-values > .22). The effect of Condition was also significant in the Autism group (F(2, 38) = 6.72, p = .003, η_p_^2^ = 0.26). Here, however, the difference between Self (M = 1.06, SE = 0.16) and Close Other (M = 0.91, SE = 0.12) was not significant (p = .37), but baseline-subtracted amplitudes were significantly larger for Self than for Stranger (M = 0.44, SE = 0.08; p = .007) and higher for Close Other than for Stranger (p = .007). Again, effects of Laterality were not significant (p-values > .09).

For the ANOVA for the frontocentral cluster, the main effect of Condition was again found to be significant (F(2, 84) = 24.46, p < .001, η_p_^2^ = 0.37). Pairwise comparisons showed larger baseline-subtracted amplitudes for Self (M = 0.68, SE = 0.06) than Close Other (M = 0.51, SE = 0.06; p = .02) and Stranger (M = 0.20, SE = 0.04; p < .001), and stronger for Close Other than Stranger (p = < .001). Here, the interaction with Group was not significant (F(2, 84) = 2.80, p = .07, η_p_^2^ = 0.06).

**Name task**

***Attention check***

Average accuracy on the attention check was 99.1%, with no significant differences between groups (t(50) = 0.69, p = .49) or conditions (all p-values > .32).

***Name discrimination***

The ANOVA for the bilateral temporo-parietal clusters revealed a significant main effect of Condition (F(2, 100) = 7.15, p = .001, η_p_^2^ = 0.13). Follow-up comparisons showed a significant difference between the Self (M = 0.42, SE = 0.05) and Stranger condition (M = 0.21, SE = 0.04; p = .001) and between the Close Other (M = 0.34, SE = 0.04) and Stranger condition (p = .02), but not between Self and Close Other (p = .14). The interaction with Group was not significant (F(2, 100) = 0.55, p = .58, η_p_^2^ = .01). There were no other significant main or interaction effects.

For the ANOVA for the frontocentral cluster, the main effect of Condition was again found to be significant (F(2, 100) = 3.63, p = .03, η_p_^2^ = 0.07). Pairwise comparisons showed greater baseline-subtracted amplitudes for Self (M = 0.17, SE = 0.03) than Stranger (M = 0.07, SE = 0.03; p = .02) and for Close Other (M = 0.17, SE = 0.03) than Stranger (p = .03), with no significant difference between Self and Close Other (p = .83). Again, the interaction with Group was not significant (F(2, 100) = 0.09, p = .92, η_p_^2^ < .01).
